# ATP and major affective disorders: the involvement of P2X receptors in pathophysiology

**DOI:** 10.1007/s11302-026-10147-5

**Published:** 2026-04-14

**Authors:** Simona Mattova, Patrik Simko, Elena Colzi, Erika Stammova, Nicol Urbanska, Maria Grigoroiu-Serbanescu, Elisabetta Coppi, Terezia Kiskova-Simkova

**Affiliations:** 1 Department of Pathology, Faculty of Medicine, Pavol Jozef Safarik University in Kosice, Rastislavova 43, 040 01 Košice, Slovakia; 2 Department of Neuroscience, Psychology, Drug Research and Child Health (NEUROFARBA), Section of Pharmacology and Toxicology, University of Florence, 50139 Florence, Italy; 3 Biometric Psychiatric Genetics Research Unit, Alexandru Obregia Clinical Psychiatric Hospital, Bucharest, Romania

**Keywords:** P2X receptors, Depression, Platelets, Bipolar disorder, Purinergic genes

## Abstract

P2X receptors, a family of ATP-gated ion channels, are increasingly recognized as key contributors to the pathophysiology of major depressive disorder. Among them, P2X7 plays a central role in stress-induced neuroinflammation by driving microglial activation, inflammasome signaling, and downstream reductions in BDNF and neuroplasticity. Additional P2X subtypes, including P2X4, further modulate neuronal and glial communication relevant to mood regulation. Evidence from animal models, human genetic studies, and early therapeutic trials supports the involvement of P2X signaling in depressive phenotypes and highlights P2X7 antagonists as promising candidates for novel antidepressant strategies. Overall, targeting P2X receptors offers a mechanistically distinct approach to understanding and treating depression.

## Depressive disorders

Depressive disorder is a common mental illness that, according to the World Health Organization (WHO), affects more than 300 million people worldwide [1]. It occurs almost twice as frequently in women as in men [2]. A clinical diagnosis relies on the identification of symptoms, as distinguishable biological markers are currently lacking. A patient with depressive disorder suffers from low mood, decreased energy, and reduced activity levels. The ability to experience pleasure is impaired (anhedonia), interest and concentration are weakened, and pronounced fatigue is common even after minimal effort. Self-esteem and self-confidence are often reduced, and even in mild cases, self-blame and feelings of worthlessness are frequent. Depressed mood typically exhibits minimal diurnal variation, remains largely unresponsive to external circumstances, and is frequently accompanied by a constellation of somatic symptoms. These may include sleep disturbances (insomnia or hypersomnia), diurnal worsening of mood, predominantly in the morning, marked psychomotor retardation or agitation, decreased or increased appetite leading to weight loss or weight gain, and decreased libido. Depending on the number and severity of symptoms, a depressive episode is classified as mild, moderate, or severe. If depressive symptoms occur repeatedly, the condition is referred to as recurrent depressive disorder (ICD 10, DSM-V classification). According to the DSM-V criteria [3] for diagnosing a major depressive episode, an individual must exhibit at least five specific symptoms almost every day for a duration of at least two weeks. These symptoms are depressed mood, reduced interest and pleasure, significant weight loss or weight gain, insomnia or hypersomnia, psychomotor agitation or retardation, fatigue or loss of energy, feelings of worthlessness or excessive or inappropriate guilt, diminished ability to think or concentrate, or indecisiveness, recurrent thoughts of death or suicidal ideation or suicide attempt. The symptoms must cause clinically significant distress or impairment in social and occupational functioning of the individual and are not the effect of another medical condition or substance abuse.

This symptom cluster often overlaps with depressive symptoms occurring in conditions such as schizophrenia and bipolar disorder. Accurate diagnosis of depressive disorder requires the application of exclusion criteria [4]. Furthermore, depressive disorder is associated with an increased risk of developing other medical conditions, such as diabetes mellitus [5], cardiovascular diseases [6], and stroke [7]. It is an extremely complex disorder with multiple causative factors, including genetic, epigenetic, and environmental influences. It involves several pathophysiological mechanisms, which can be categorized into neurochemical changes, tissue- and organ-level pathologies such as inflammation and an increased stress response, as well as alterations in neural circuitry [8]. Given the significant limitations that mental disorders impose on quality of life and their high prevalence in the human population, it is essential to seek effective treatment strategies. Currently, treatment primarily focuses on monoamine reuptake inhibitors, including those targeting norepinephrine, dopamine, and 5-hydroxytryptamine (5-HT) [9]. However, these approaches appear to be insufficient. More than 50% of patients fail to achieve an adequate response to their initial antidepressant treatment [10]. In addition, the optimal efficacy of these medications is typically observed only after a considerable delay, usually between 4 and 6 weeks [11]. This highlights the urgent need to explore novel mechanisms that can further elucidate the pathophysiology and pharmacology of depression.

## Major affective disorders: bipolar disorder and unipolar major depression

Bipolar disorder (BD) and unipolar major depression (MDD-UP) are the principal major affective disorders with an important genetic component and familial transmission risk. Between BD and MDD-UP, there are important differences regarding the genetic contribution: the genetic component for BD is estimated at 0.85 and the environmental contribution at 0.15 [12, 13], while the genetic component of MDD-UP diagnosed according to DSM-IV criteria is 0.43 and the environmental contribution is 0.57 [14]. All humans carry a certain number of genetic risk factors for MDD [15]. The prevalence of MDD-UP (DSM-IV criteria) in the general American population was estimated at 16.2% [16], while in 1982, the prevalence of MDD-UP was estimated at 3% in the same population [17]. There are socio-cultural differences in the prevalence of MDD-UP. Kessler and Bromet [18] evidenced different prevalences of DSM-IV major depression (MDD-UP) in different countries from Europe, the US, Asia, and Africa (South Africa), with a mean total prevalence in high-income countries of 14.4% (range 9.9%−14.1% in Europe; 19.2% in the US) and 11% in low-middle income countries.

The lifetime prevalence of BD in the general population is 0.5%−1.5% [19]. BD is a severe, recurrent psychiatric disorder that consists of alternating episodes of mania and depression in the BD-I subtype and hypomania and depression in the BD-II subtype. The genetic correlation between mania and depression is 0.65 [12]. The BD episodes considerably affect individual functioning. The two BD subtypes have specific genetic associations [20]. BD-I has the highest genetic correlation with schizophrenia (SCZ), while BD-II has the highest correlation with MDD-UP. The SNP heritability (h^2^) (h^2^ = the variance attributable to genome-wide SNPs) of BD-II is lower (h^2^_SNP_ = 11%) than the SNP heritability of BD-I (h^2^_SNP_ = 21%) [21].

The prevalence of BD-I is 0.6%, and the prevalence of BD-II is 0.4% in the world population [22]. There are no significant sex prevalence differences.

Purinergic signaling, which involves extracellular adenosine acting through P1, and adenosine 5'-triphosphate (ATP) acting through P2 receptors, is increasingly recognized as an important factor in the development of depression. This signaling system influences several key processes in the brain, including neuroinflammation, synaptic plasticity, and the regulation of neurotransmitters [11]. These effects occur mainly in brain regions involved in emotional and cognitive processing, such as the hippocampus, prefrontal cortex, amygdala, and striatum [23, 24]. In a similar way, disturbances in purinergic signaling have also been linked to the pathophysiology of BD. In this condition, ATP, adenosine, and their receptors contribute to mechanisms related to neuroinflammatory responses, cellular energy regulation, and neurotransmission [25]. Alterations in this system are particularly evident in brain areas responsible for mood regulation and cognitive functions, including the hippocampus, prefrontal cortex, striatum, and nucleus accumbens [26].

Diverse evidence from rodent models of MDD corroborates the close relationship between astrocyte purinergic signaling and depression. For example, mice exposed to chronic social defeat stress (CSDS) show decreased ATP levels, especially in brain slices from the prefrontal cortex and hippocampus [27, 28]. Within the brain, many P2X and P2Y receptors are expressed in the hippocampus, where they participate in the regulation of glutamate release [29]. Dysregulation of neurotransmitter systems in the central nervous system (CNS) is considered one of the key mechanisms underlying depression [30]. In particular, excessive glutamate release and subsequent overactivation of NMDA receptors are closely associated with depressive pathology, as they can induce excitotoxicity and lead to neuronal injury [31, 32]. Numerous studies, especially those focusing on the hippocampus, have demonstrated that activity-dependent modifications in the strength of glutamatergic synapses are crucial for learning and memory formation, and disruption of these processes can impair memory consolidation [33–35]. Additionally, chronic and intense stress has been shown in animal models of depression to negatively affect hippocampus-dependent explicit memory [36]. This impairment is thought to result from alterations in hippocampal synaptic plasticity, specifically in mechanisms such as long-term potentiation (LTP) and long-term depression (LTD), where severe stress tends to suppress LTP while promoting LTD [30].

## P2X receptors

Recently, there has been a growing interest in understanding the role of the purinergic system in the CNS [11]. P2X membrane receptors represent a family of cation-selective ion channels, with seven P2X receptor (P2XR) subunits identified (P2X1–7) [37]. These receptors exhibit a broad tissue distribution. They have been found in a wide range of cell types, including neurons and glial cells in both the CNS and peripheral nervous systems (PNS), as well as in muscle, bone, epithelial, endocrine, endothelial, and immune cells [38]. The cell surface P2X receptors are activated by ATP [39]. ATP has long been recognized as the primary energy source in living cells. However, a significant additional role of ATP has since been discovered: extracellular ATP (eATP) acts as a messenger, facilitating rapid intercellular communication by binding to and activating a group of membrane proteins known as P2X and P2Y receptors. Unlike traditional neurotransmitters and neuromodulators, the extracellular effects of ATP are pleiotropic, influencing nearly all cell types throughout the body [40]. P2X receptors mediate ATP signaling primarily through three mechanisms: they function as ligand-gated, Na^+^, K^+^, and Ca^2^⁺-permeable cation channels, promote the formation of membrane macropores, and form signaling complexes with interacting proteins and membrane lipids [38]. ATP-mediated signaling via P2X receptors plays a critical role in various essential physiological processes, including neurotransmission, muscle contraction, and cytokine release [39]. They are also involved in pathophysiological processes in the body due to altered expression and function of the receptor, which can result in chronic pain, inflammatory diseases, cancer, and other pathological conditions or disorders [37]. In the context of the development and treatment of depressive disorders, only a few P2X receptor subtypes have been investigated, primarily P2X4 and P2X7 [11]. Studies suggest that P2X7 receptor blockade induces antidepressant effects [41, 42]. Studies also suggest antidepressant effects associated with the P2X4 receptor [43, 44]. Current research in this area supports the involvement of P2X receptors in major affective disorders.

## P2X7 receptors

Strong evidence of the involvement of the P2X7 receptor (P2X7R) in major affective disorders has been described. P2X7R is assembled as a homo-trimeric channel, each subunit being composed of two transmembrane helices and a large extracellular domain, with the ATP binding site in an inter-subunit pocket [45]. The long C-terminal cytoplasmic domain contains binding sites for Zn^2^⁺ and presumably GDP, suggesting a role in intracellular signaling pathways, beyond the P2X-mediated regulation of neurotransmission [46]. Another peculiarity of the P2X7R is its low affinity for the endogenous ligand, as it requires extracellular ATP concentrations above 100–300 μM, achieved exclusively during tissue damage or pathological conditions [47]. For this reason, the P2X7R is considered a sensor of damage, and the ATP/P2X7 pathway is listed among the damage-associated molecular patterns (DAMPs) [48]. Like other P2X receptors, the P2X7R is permeant to Na⁺, Ca^2^⁺, and K⁺ ions and, upon activation, produces an inward current generating a depolarization of the cell membrane and an increase in intracellular Ca^2+^. Unlike all other P2X receptors, little or no desensitization is observed for the P2X7-mediated current upon agonist (ATP or the synthetic compound benzoil-ATP exposure, which might generate excessive Ca^2+^ influx and cytotoxicity [49]. Of note, in case of prolonged ATP exposure or excessive concentration, the receptor evolves into a "large-pore" ATP-gated channel permeable to molecules up to 900 Da, including pro-inflammatory cytokines, thus contributing to neuroinflammation and cell death [50, 51].

P2X7R stimulation is associated with the activation of the NLR Family Pyrin Domain Containing 3 (NLRP3) inflammasome and consequent release of pro-inflammatory interleukin (IL-) IL-1β and IL-18, and activation of pro-apoptotic Caspase-1 pathway [50, 52], thus boosting inflammatory processes. Furthermore, P2X7R activation mediates the release of membrane-delimited extracellular vesicles (EVs), microvesicles, and other microparticles as a mechanism for intercellular communication during the inflammatory response [53]. Of note, this is among the mechanisms by which the P2X7R, overexpressed in cancer cells, contributes to metastasis dissemination [54], thus adding this particular mechanism to the body of evidence indicating the P2X7R as a target for innovative antitumoral strategies [55].

The P2X7R is widely expressed on immune cells like monocytes/macrophages, dendritic cells, and T cells [52], where its activation promotes their conversion to the activated phenotype throughout the above-mentioned NLRP3/caspase-1 inflammasome complex induction. Within the brain, it is abundantly expressed on microglia, the resident immune cells of the CNS, oligodendrocytes, and ependymal cells [56]. Conversely, functional P2X7R expression in neurons is still under debate [57]. Such controversies could be due to brain region- or developmental stage-specific changes in receptor expression, as well as to the induction of neuronal expression under specific pathological conditions [58]. Of note, recent data confirmed neuronal P2X7R expression in hippocampal hilar interneurons, where their effects might diverge from those mediated by the same receptor expressed on microglia, as demonstrated in epileptic mice [59].

Nowadays, a very important relationship is discussed indeed. Interactions between the gut microbiota and the host immune system are complex, dynamic, and dependent on the host. Recent research indicates that disruptions in the gut bacterial community can reduce the production of certain metabolites, which may in turn contribute to behavioral changes such as depression [60]. *Nervus vagus*, which is responsible for facilitating communication, provides support for the microbiota-gut-brain axis [61]. P2X7R is a key component in these pathways, specifically acting as a receptor for ATP that, when activated by stress-induced damage, triggers inflammatory responses linked to depressive symptoms. It has been shown that the NLRP3 inflammasome and upstream P2X7 receptor are biological substrates for depression [62]. Activation of P2X7R by stress or inflammatory signals initiates the NLRP3 inflammasome, leading to the release of pro-inflammatory cytokines such as IL-1β and IL-18, which are associated with depression [63]. Influencing the inhibition of P2X7R/NLRP3/IL-1β expression, for example, via electroacupuncture, may lead to alleviation in depression-like behaviors in experimental rats [64].

## P2X7 receptors and microglia activation in major affective disorders

Microglial activation is a hallmark of neuroinflammation occurring in several neurodegenerative diseases, such as Alzheimer’s and Parkinson’s diseases or multiple sclerosis [65, 66]. In the psychiatric disorder schizophrenia, P2X7Rs may participate in the modulation of granular cell excitatory neurotransmission in the dentate gyrus via EC-GC pathway, contributing to pathological alterations of neuronal functions leading to neurodevelopmental disorders [67]. However, another study did not find any association between P2X7R and schizophrenia, although it may be involved in several neuronal processes associated with schizophrenia [68].

Similarly, dysregulation in microglial cells has recently been raised as a possible mechanism contributing to the pathophysiology of depression [69], as suggested by preclinical and clinical studies where pro-inflammatory cytokines or endotoxins induced depressive-like symptoms in animal models or human volunteers [70]. Accordingly, studies reported morphological and structural microglial alterations in the CNS of depressed rodent models and *postmortem* tissue of the patients [71].

Many chronic systemic inflammatory states, e.g., diabetes, rheumatoid arthritis, inflammatory bowel disease, and chronic liver disease, present high comorbidity with depression [72, 73], suggesting that stress may be causally related to the pathophysiology of depression, as recently postulated [74]. Accordingly, both acute and chronic stress have been documented to induce dramatic changes in the number, morphology, and functions of microglia [75]. This stress-induced microglial activation might induce direct or secondary alterations in neurotransmission through impaired synaptic plasticity and pruning, inhibiting neurogenesis and elevating neurotoxicity [76].

The microglia–P2X7–inflammasome axis is a critical neuroinflammatory pathway wherein high levels of eATP bind to P2X7 receptors (P2X7R) on microglia, triggering the activation of the NLRP3 inflammasome. This process causes the maturation and release of pro-inflammatory cytokines, specifically IL-1β and IL-18, driving neuroinflammation and, in extreme cases, microglial pyroptosis [62, 77, 78]. In this context, the P2X7R might be a key interface between stress-induced microglial alterations and the appearance of depressive-like symptoms, i.e. through NLRP3 inflammasome activation and consequent unbalanced pro- *vs* anti-inflammatory milieu and/or altered neurotransmission [79] as recent evidence demonstrates that microglial P2X7R engagement in rodents exposed to chronic unpredictable stressors, known to contribute to depression, increases eATP and activates the NLRP3 inflammasome in the hippocampus [80]. Importantly, in the same work, the authors showed that genetic ablation or pharmacological inhibition of P2X7R prevented the development of depressive-like behaviors.

Another mechanism by which P2X7Rs might contribute to depressive-like behaviors in mice has been recently postulated by Zhang and co-authors [81], based on the alterations in mitochondria-associated membranes (MAMs) induced by excessive eATP in microglial cells in mice exposed to chronic social defeat stress (CSDS). The authors postulate that mitochondrial stress and MAM modifications, alongside facilitated Ca^2+^ transport between the endoplasmic reticulum (ER) in hippocampal microglia, are concomitant with the appearance of depression-like symptoms in CSDS mice. Additionally, exposing microglia to eATP to mimic CSDS conditions resulted in analogous outcomes. Hence, the authors conclude that CSDS augments eATP, which, by activating the P2X7R, induces ER stress, mitochondrial damage, and NLRP3 inflammasome aggregation, thus facilitating communication between the ER and mitochondria in microglia subtype, thereby contributing to the development of depression-like phenotypes in male mice [81].

Interestingly, adenosine kinase (ADK) is upregulated in epilepsy and depression and has been implicated in promoting chronic unpredictable stress-induced neuroinflammation and depressive-like behaviors in mice [82].

Chronic sleep deprivation is considered another substantial risk factor for major depressive disorder [83] and induces depressive-like behaviors in animal models [84]. This condition promotes a gradual elevation of eATP in the brain of sleep-deprived mice, leading to the activation of the P2X7/NLRP3/Caspase-1 pathway; depressive-like symptoms developed by these animals were alleviated in P2X7R-null littermates [85].

Worth noticing, P2X7R-null mice present, per se, an antidepressant-like profile when tested on multiple behavioural paradigms (i.e., forced swim test: FST; tail suspension test: TST; elevated plus maze, novelty suppressed feeding, spontaneous locomotor activity, and food intake) and higher responsivity to a subefficacious dose of the antidepressant drug imipramine (15 mg/kg) [86]. Data are consistent with further evidence demonstrating a reduced immobility in the TST and decreased behavioural despair in the FST in P2X7R-deficient mice, as well as a decreased sensitivity to amphetamine-induced hyperactivity [87]. In ìthe same work, Sperlagh and co-authors demonstrated that basal norepinephrine levels were elevated in the amygdala, whereas stress-induced corticosteroid responses were alleviated in P2X7R-null mice and, of note, a sub-acute treatment with the selective P2X7R antagonist, Brilliant Blue G, reproduced the effect of genetic deletion in the TST test and amphetamine-induced hyperactivity in wt animals, further strengthening the prodromal facilitatory role of P2X7Rs in depressive-like behaviors. Another piece of evidence comes from the inescapable footshocks test, which leads to learned helplessness behavior measured as an increased latency and number of escape failures to subsequent escapable footshocks. This behavior is accompanied by downregulation of mRNA encoding P2X7Rs and decreased number of spine synapse density in the dentate gyrus [88]. Strikingly, no learned helplessness behavior is observed in P2X7R-deleted mice, nor a decrease in spine synapse number in response to inescapable footshocks, demonstrating that endogenous activation of P2X7Rs in the learned helplessness model of depression causes decreased plasticity of spine synapses, whereas, in P2X7R-deficient mice, preserved synaptic plasticity and escape behavior to repeated stressful stimuli are observed. Finally, recent evidence from Ribeiro and co-workers was released to confirm the outstanding antidepressant-like effects exerted by P2X7R blockade [89]. By taking advantage of the Flinders Sensitive Line (FSL) rat model, the authors demonstrate that repeated treatment with the P2X7R antagonist A-804598 (30 mg/Kg) reduced the immobility time in the FST by enhancing BDNF-mediated signalling in the ventral hippocampus, thus supporting the notion of P2X7R antagonism as a potential new antidepressant strategy. In summary, outstanding evidence demonstrates a pro-depressive role of P2X7R activation in the etiology of depression. This knowledge may shed light on the development of novel P2X7-targeted intervention strategies against this devastating mental health issue.

## P2X7 antagonists as promising candidates for novel antidepressant strategies

Targeting the P2X7R with antagonists shows significant promise as novel antidepressants by blocking excessive ATP-driven inflammation, microglial activation, and cytokine release triggered by stress, which are key factors in depression [90]. Within the neuroinflammatory mechanisms associated with depression, the P2X7R-dependent NLRP3/IL-1β signaling pathway plays an important role in the activation of the innate immune system [91]. P2X7R antagonists, therefore, represent a promising new class of antidepressants, as they suppress neuroinflammation by inhibiting activation of the NLRP3 inflammasome and decreasing levels of pro-inflammatory cytokines such as IL-1 [92]. Various studies have used P2X7R antagonists, such as Brilliant Blue G (BBG), A-438079, and A-804598, in CPSS/CPPS-based murine models of depression, resulting in the mitigation of neurobiological and behavioral alterations caused by stress [42, 80, 93, 94]. Some indices show that other P2XR antagonists may also show antidepressant effects; they have not primarily been developed and tested so far for this purpose.

Clinical studies have shown that JNJ-54175446 possesses favorable pharmacokinetic and safety profiles, along with notable anti-inflammatory properties, as demonstrated in a randomized single ascending dose study conducted in healthy volunteers [95, 96]. More recently, a 2023 study reported that JNJ-54175446 attenuated the acute dysphoria induced by total sleep deprivation (TSD), supporting its potential as a candidate for antidepressant therapy [97]. Another P2X7 receptor antagonist, JNJ-55308942, was evaluated in a Phase I clinical trial in 2017 to assess its safety in healthy participants. Subsequently, the compound advanced to a Phase II clinical trial in 2022 to investigate its efficacy in patients with bipolar depression, as reported in the ClinicalTrials.gov database, as reviewed in [90].

## P2X4 receptors

Emerging evidence suggests that the P2X4 receptor (P2X4R) plays a role in major affective disorders. P2X4R was first isolated from cDNA of rat brain in 1996 [98]. Since then, it has been identified in various human tissues. Within the CNS, P2X4R is found in neurons and microglia [99]. In the PNS, they are also widely expressed [100]. Moreover, they are abundantly present in cells of the immune system, including T lymphocytes, B lymphocytes, macrophages, mast cells, and eosinophils [101]. Regarding other sites of expression, P2X4R has also been identified in the pancreas and salivary glands. Additionally, they are present in the gastrointestinal tract (GIT) [102], kidneys, lungs, bladder, and the endometrial tissue of the uterus [103]. In the cardiovascular system, they play an important role. On one hand, they act as negative regulators of heart rate, while on the other, they serve as positive regulators of cardiac contractility by their cation-exchange mechanism [104].

Molecular structure of P2X4R is of a trimeric channel, which was confirmed by solving the crystal structure of a zebrafish P2X4R in 2009 during ATP-unbound closed state [105] and later during ATP-bound open state in 2012 [106]. As it is composed of three identical subunits, each subunit has a structure that can be compared to a leaping dolphin. Two transmembrane domains of each subunit for the tail anchor the receptor to the cell´s lipid membrane. The extracellular loop and head domain form a body and are projected into the extracellular space, where they can bind to extracellular ATP. These subunits may vary in their electrophysiological and/or pharmacological characteristics [107]. P2X receptors also differ in their sensitivity for ATP and desensitization kinetics, with the P2X4R exhibiting intermediate ATP sensitivity (EC_50_** = **3–10 µM) and moderate desensitization [107]. Ca^2+^ current measurement showed its highest permeability for Ca^2+^ ions among all P2X receptors [108]. Under normal conditions, the P2X4R is typically inactive [100]. Within the cell, it is localized predominantly in lysosomes and endosomes, where it is stored and subsequently transported to the cell surface if needed, for example, in response to various signals such as inflammatory mediators. After reaching the cell surface, the receptor is sensitive and ready for ATP binding. When extracellular ATP binds to the receptor, it causes a conformational change that opens the ion channel [109]. When opened, the P2X4R is highly permeable for cations, particularly Na^+^ and Ca^2+^, which enter the cell and trigger a series of intracellular signaling cascades, depending on the cell type involved. Concomitantly, with an influx of Na^+^ and Ca^2+^ to the cell, we see an exflux of K^+^ ions from the cell [110]. For example, this Ca^2+^ influx in microglial cells stimulates the release of brain-derived neurotrophic factor (BDNF), and thus neuronal excitability, contributing to altered neuronal signaling and potential pathophysiological changes in the nervous system [111]. Activation of P2X4R also induces the release of prostaglandin E_2_ (PGE_2_) from macrophages, further contributing to inflammatory and immunomodulatory processes, such as neurodegenerative disorders, like Alzheimer's and Parkinson´s diseases, and depression, when elevated levels of PGE_2_ were found in cerebrospinal fluid and serum of depressed patients [112]. Therefore, P2X4R may present a promising therapeutic target for the treatment of depression [113]. P2X4R is further implicated in mood and depression via its role in neuroinflammation, particularly the activation of microglia in the hippocampus [114]. The hippocampus is a brain structure localized in the medial part of the temporal lobe and plays a key role in mood regulation and memory, and is very sensitive to stress factors. When a depressive condition occurs, there is an atrophy of hippocampal mass, including a reduced count of neurons [115]. P2X4R on the microglial membrane is activated by extracellular ATP, which is released from damaged cells. Chemokines such as CCL2 and CCL21 then increase the presence of P2X4R on the cell surface by its trafficking from intracellular compartments [116]. This activation leads to the release of proinflammatory cytokines like IL-1 and the activation of NLRP3 inflammasome, which further contribute to the depressive-like behavior [117]. On the other hand, P2X4R activation could promote a release of BDNF, which has antidepressant effects and suggests a dual role of P2X4R in mood regulation, that is dependent on the time after an injury or the specific brain region involved [118]. For example, in the recovery phase after a stroke, activating P2X4R in microglia can release BDNF, which is a physiological response to the brain damage and alleviates its impact on the tissue [119]. However, in the model of depression, P2X4R activation in microglia can contribute to synaptic dysfunction. The P2X4 antagonist 5-BDBD reversed abnormal changes in the hippocampus, relieved hippocampal neuronal damage, and alleviated the abnormal pain and depressive-like behaviors in rats with comorbid chronic pain and depression [113].

## P2X4 antagonists as promising candidates for novel antidepressant strategies

P2X4R antagonists may show promise in depression research, especially for comorbid chronic pain and depression [113, 120], by blocking microglial activation, reducing pro-inflammatory cytokines (like IL-1β), and preventing neuronal damage (pyroptosis) in the hippocampus [120]. While P2X4R activation can drive depression-like behaviors (e.g., after stroke), blocking it with agents like 5-BDBD helps alleviate depressive symptoms, suggesting P2X4R as a target for new potential antidepressants, particularly in pain-related mood disorders [113].

Also, other antagonists of P2X receptors are being explored, but for other targets and have not been fully tested for depressive disorders or symptoms.

## P2X2 receptors

An increasing amount of data discusses the possible involvement of the P2X2 receptor (P2X2R) in major affective disorders. However, its direct role has not been confirmed yet. P2X2R is another ATP-gated ion channel, primarily consisting of a large extracellular domain for ATP binding, two transmembrane domains (TM1 and TM2) for each subunit, and intracellular N- and C-termini. Like other above-mentioned P2X receptor subtypes, the P2X2R forms a pore permeable to Na^+^, K^+^, and Ca^2+^ ions [121]. Functionally, it plays a role in neurotransmission, particularly fast synaptic transmission between neurons and smooth muscle, and is involved in physiological processes like hearing [122]. Thus, P2X2Rs play an important modulatory role in neuronal excitability, synaptic transmission, and neuroinflammatory signaling. Although research on P2X2R is less extensive than on other purinergic receptors, accumulating evidence suggests that P2X2R may contribute to the pathophysiology of depressive disorders through several interconnected mechanisms [123, 124].

It is well known that ATP regulates a number of behaviors, involving mood and motivation, learning and memory, sleep and arousal, locomotor, feeding activities, and cognition, via P2X ligand-gated cationic or G protein-coupled receptors (P2YRs) [125]. As already described before, P2X receptors are involved in all those processes. However, some new studies link the activity of P2X2R to the modulation of mood and thus to some mood and stress-related disorders. P2X2Rs are expressed in brain regions critically involved in mood regulation, including the hippocampus, prefrontal cortex (PFC), and amygdala. Activation of P2X2Rs influences glutamatergic and GABAergic neurotransmission and modulates LTP [126]. Dysregulation of these pathways has been linked to impaired synaptic plasticity, a hallmark biological feature of depression. For example, Kuang et al. (2022) have shown that P2X2R levels were increased in the medial prefrontal cortex (mPFC) of depression-susceptible mice, and selective knockout and overexpression of P2X2R in mPFC pyramidal neurons bidirectionally regulated depressive-related behaviors. Furthermore, the study demonstrates that P2X2Rs in mPFC pyramidal neurons regulate vulnerability to chronic stress [124].

As mentioned above, chronic stress is described as one of the major risks for depression and may alter purinergic signaling [127], with the hypothalamic–pituitary–adrenal (HPA) axis playing a central role in mediating stress responses [128]. The lack of P2X2Rs promotes resilience to stress-induced depressive-like behaviors. Stress-induced ATP release may lead to abnormal P2X2R activation, contributing to neuronal dysfunction. It was found that P2X2R in pyramidal neurons is sufficient and necessary to regulate neuronal plasticity in the mPFC and depressive-like behaviors. These findings identify the regulation of excitatory synapses onto CamkIIα mPFC neurons by P2X2R as an important mechanism underlying mood disorder, representing a potential medicinal target for MDD [124].

It has been shown that among all, P2X2Rs may also be involved in inflammatory signaling; however, only indirectly. In humans, alveolar macrophages were reported to express all P2 receptor subtypes except P2Y12R, P2X2R, P2X3R, and P2X6R [129].

Zheng et al. (2023) revealed a significant relationship between P2X2 mRNA expression and depression, and they confirmed a relationship between P2X2R, epoxide hydrolase gene (EPHX2), and MDD in humans and presented preliminary haplotype-based evidence that implicates EPHX2 in suicide [130].

Taken together, the role of P2X2R in depression involves a neurophysiological and circuitry-modulating role in depression.

## Involvement of other P2X receptors in major affective disorders

**Fig. 1:**
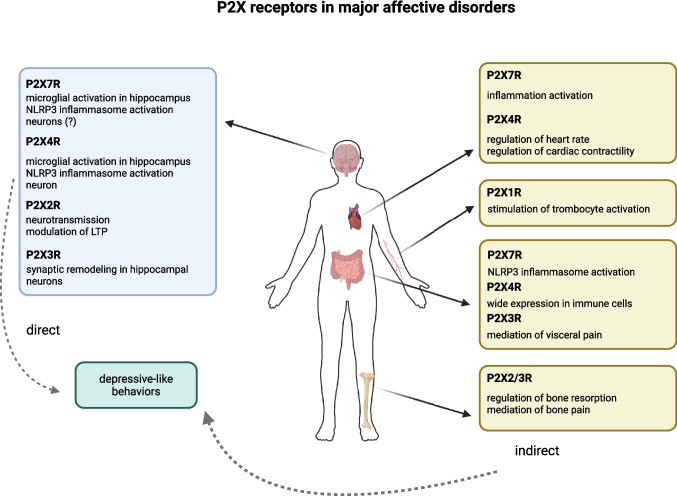
The various roles of P2X receptors in major affective disorders, leading to depressive-like behaviors. Created in BioRender

While the direct role of P2X7Rs and P2X4Rs in MDD and BP has already been described, P2X2Rs are currently being discussed within the scientific community. The involvement of other receptors from this receptor group in major affective disorders remains indirect or speculative (Fig. 1).

## P2X3 receptors

The P2X3 receptor (P2X3R) is a trimeric ATP-gated ion channel predominantly expressed in small- to medium-sized nociceptive neurons of the dorsal root and trigeminal ganglion, where it mediates fast synaptic transmission of pain signals [131]. It forms either homotrimers (P2X3R) or heterotrimers (P2X2/3R), both permeable to Na⁺, K⁺, and Ca^2^⁺ ions. Structurally, the receptor exhibits rapid activation and desensitization kinetics in response to a nanomolar concentration of eATP. Heteromeric assemblies such as P2X2/3R demonstrate slower desensitization and are involved in prolonged sensory signaling [132]. Under inflammatory and neuropathic conditions, P2X3R becomes upregulated or sensitized, which contributes to chronic pain and sensory hypersensitivity [133, 134]. P2X3R signaling also contributes to visceral sensitivity, particularly in organs such as the gastrointestinal tract, where it mediates discomfort and pain associated with disorders like overactive bladder or irritable bowel syndrome [135, 136]. Additionally, P2X3R participates in neuro-immune communication, as ATP-mediated activation modulates inflammatory processes in peripheral tissues and influences interactions between sensory neurons and immune cells [136]. Emerging findings further suggest that stress can alter P2X3R function, linking increased receptor activity to stress-related somatic symptoms and potentially to mood-associated physiological changes [137, 138].

Although P2X3R is best known for its role in nociception, several studies suggest they may also participate in mechanisms relevant to depression. Early evidence comes from synaptic plasticity research, where P2X3R knockout mice were shown to exhibit impaired LTD while maintaining normal LTP, indicating that ATP-dependent P2X3R signaling contributes to forms of synaptic remodeling in hippocampal neurons implicated in mood regulation [139]. Additional insight comes from findings that P2X3R interacts with the calcium/calmodulin-dependent serine protein kinase (CASK), which modulates receptor stability and surface expression; such modulation of neuronal plasticity may influence stress-related neuronal responses [140]. More recent work shows that inflammatory mediators, including TNF-α, can enhance P2X3R expression and ATP sensitivity in sensory neurons, linking P2X3R signaling to neuroimmune pathways often dysregulated in comorbid depression with chronic pain [23]. By increasing neuronal excitability and mediating pain-related sensory input, P2X3R activity may influence mood. As a result, modulating this receptor is being explored as a way to reduce chronic pain and the depressive symptoms that often accompany it [141]. Taken together, these findings suggest that although P2X3R does not appear to function as a core mediator of affective regulation, its involvement in synaptic plasticity mechanisms, neuroimmune activation, chronic pain, and stress-related somatosensory pathways may indirectly contribute to the development or exacerbation of depressive symptomatology, particularly in individuals with co-occurring chronic pain or sustained inflammatory states.

## P2X1 receptors

The P2X1 receptor (P2X1R) is characterized by high affinity for the endogenous ligand ATP and rapid desensitization following activation [142]. It exhibits relatively high permeability to Ca^2^⁺ ions [108] and plays a key role in neurogenic smooth muscle contraction, thrombus formation, and the regulation of neutrophil migration [143]. P2X1Rs expressed on platelets contribute to platelet activation and aggregation, thereby playing a significant role in the pathophysiology of thrombosis [144].

P2X1R subunits can form both homomeric and heteromeric ion channels with distinct properties. Homomeric P2X1R forms ion channels in platelets and megakaryocytes. They exhibit significant Ca^2^⁺ ion permeability [145]. In vitro studies report that selective activation of P2X1Rs induces a rapid and reversible change in platelet shape through an increase in intracellular Ca^2^⁺ levels [146]. Activation of P2X1Rs leads to Ca^2^⁺ and Na⁺ influx and can also result in platelet membrane depolarization. This receptor also supports transient granule centralization and stimulates a low level of αIIbβ3 integrin activation, leading to weak and transient platelet aggregation [147]. As reported by in vivo studies, P2X1R represents a potentially significant target for antithrombotic therapy [148] as it can enhance platelet activation in response to various agonists, thereby playing a role in thrombus formation [145]. ATP is released during activation from dense granules of platelets, activated endothelial cells, and leukocytes, as well as passively from dying cells during tissue injury [149]. In addition to ATP, P2X1Rs can also be stimulated by several related compounds released into the bloodstream, including various diadenosine polyphosphates (ApnA) and adenosine polyphosphoguanosines (ApnG). These receptors can also act synergistically with a variety of other receptors to enhance signaling and functional activities in platelets. Activation of P2X1Rs by ATP released from dense granules potentiates aggregation responses to low levels of major agonists, such as thrombin and collagen [145] or adrenaline [150].

Patients with depression exhibit increased platelet activation and reactivity, and treatment of depressive symptoms is associated with normalization of platelet activation markers. The mechanisms underlying increased platelet activation in depression remain unclear. Apart from the potential role of serotonin, one possible explanation is that depression may overlap with anxiety and panic attacks, which can lead to excessive sympathetic activity. In such cases, platelets could respond to adrenaline or noradrenaline released during stress [151]. So far, no research has directly examined the association between P2X1Rs in blood and depression.

## Genetic studies of major affective disorders and purinergic genes

It is hard to imagine medical practice without the expansive knowledge of genetics and genomics that has been accumulated over decades [152]. And indeed, studying major affective disorders is no exception. Almost 40–50% of MDD and 60%–90% of BD have been found to be heritable [153]. Genome-wide association studies (GWAS) have identified over 100 common variants associated with depression [154]. In relation to purinergic signaling, the first study of the P2X7R gene in major affective disorders was a genome-wide linkage study of large French-Canadian families from the Province of Québec, multiply affected with BD [155].

Since then, several studies have applied the candidate gene approach, specifically focusing on the genotyping of selected single-nucleotide polymorphisms (SNPs), primarily within the P2X7R gene. This gene, located on chromosome 12q24, encodes a purinergic receptor expressed in the brain and participates in Ca^2^⁺-dependent signaling pathways [156]. The chromosomal region 12q23–q24.1 has been suggested as a potential susceptibility locus for affective disorders based on several linkage and association studies. In this context, Ewald et al. (1998) investigated the possible linkage between bipolar affective disorder and 16 microsatellite markers spanning chromosome 12q22–q24 in two Danish families. Their analysis revealed that an overlapping segment of chromosome 12q24 was shared by all but one of the bipolar patients, although the haplotypes differed between the two families [157]. Another seven families were selected based on containing multiple cases of BD present in three or more generations, an absence of schizophrenia, and unilineal transmission. The results show strong support for linkage to the region of 12q23-q24 around D12S342. Moreover, the region of 1q and 1p close to D1S243 may also harbor susceptibility genes [158]. In the study of Green et al. (2009), two pedigrees segregating both bipolar disorder and Darier's disease were studied. The non-synonymous SNP rs2230912 (located in exon 13 of the P2X7R gene) was the only SNP in the P2X7R gene linked to MDD. This SNP rs2230912 (resulting in amino-acid polymorphism Q460R), showed the strongest association and has been postulated to be pathogenically relevant [159].

Epidemiological studies indicate that environmental factors are strongly associated with the risk of developing MDD and other stress-related disorders, as reviewed in [160]. Epigenetic environmental factors in major affective disorders include childhood trauma, chronic stress, or social factors like migration, which alter gene expression without changing DNA [161]. These factors trigger lasting neurobiological changes, such as DNA methylation and histone modification, affecting neuroplasticity, neuroinflammation, and neurotransmission (e.g., serotonin/dopamine dysfunction) [160, 162]. Interestingly, P2RX7 variation may mediate the effect of early childhood adversities and traumas on later emergence of suicide risk, as reported in the study from Kristof et al. (2024). In the study, 1644 participants were asked to complete the questionnaire assessing childhood adversities, recent negative life events, and provided information about previous suicide attempts and current suicide risk-related markers, including thoughts of ending their life, death, and hopelessness. Subjects were genotyped for 681 SNPs in the P2RX7 gene, 335 of which passed quality control and were entered into logistic and linear regression models. Two significant clumps were identified with a main effect on current suicidal ideation with top SNPs rs641940 and rs1653613. In interaction with childhood trauma, a clump with top SNP psy_rs11615992 was revealed, and another clump on hopelessness containing rs78473339 as index SNP [163].

## Candidate gene studies

Contradictory results have also been reported in BD studies involving genotyping of the P2X7R gene. While using pedigrees from a French-Canadian population, showing a significant association (P value = 0.000708) of the SNP rs2230912 (P2X7R-E13A) with BD [164], three linkage studies of BD (a London-based sample of 604 bipolar cases and 560 controls) showed a strong association between BD and the markers rs2230912 (*P* = 0.043) and a microsatellite marker NBG6 (*P* = 0.010) [165]. However, a study from Vereczkei et al. (2019), studying 315 patients (195 MDD, 120 BD) and 406 healthy control subjects, did not find any association between P2RX7 gene variants and depression [166]. An analysis of some current data from Romania, Germany, Poland, and Russia, all genotyped at the Institute of Human Genetics of Bonn (1,445 BD-I patients, 640 recurrent MDD-UP patients, and 2,006 healthy controls), showed no allelic or genotypic association between rs2230912 and BD or Mdd-UP both in the national samples and in the combined European patient sample [167]. Inconsistent findings were also reported in studies of the German population. While some case–control studies identified an association between the SNP rs2230912 and MDD-UP in German samples [168, 169], another study failed to detect such an association [167]. Similarly, Green et al. (2009) analyzed a large UK cohort comprising 687 BD patients, 1,036 MDD-UP patients, and 1,204 controls and found no association between nine SNPs in the P2X7R gene, including rs2230912, and either BD or MDD-UP [159].

A singular result was reported by Backlund, Lavebratt [170] who found a significant association between the A-allele of the SNP rs2230912 with rapid cycling BD-I (121 BD-I patients compared to 446 non-rapid cycling BD-I cases and 1,044 controls) (OR = 2.2, *P* = 0.002). Rapid cycling BD is a more severe form of BD-I with at least 4 episodes per year. This result was no longer replicated.

Vereczkei, Abdul-Rahman [166] genotyped the P2X7R-SNP rs2230912 and 5 other non-synonymous SNPs around 7 kb from this SNP in 195 MDD-UP cases, 120 BD cases, and 406 healthy controls from Hungary. Haplotype analysis indicated a relatively high linkage between the analyzed P2X7R SNPs. No association between P2X7R gene variants and depression was detected at the allelic or genotypic level. Instead, a significant association of the depression symptom severity measured with the Hamilton Depression Rating Scale and the SNPs rs2230912 (Gln460Arg) and rs1718119 (Ala348Thr) of the P2RX7 gene was observed in BD patients (nominal, not corrected *P*-values: *P* = 0.008 for SNP rs1718119 and *P* = 0.013 for SNPs rs2230912), but not in MDD-UP patients. This result was not replicated.

Feng, Zhang [171] performed a meta-analysis of all studies published by that date on the P2X7R gene in affective disorders, including all subjects used in the above-mentioned studies. They found no significant association of the P2X7R-SNP rs2230912 with BD (6,514 BD cases and 11,254 controls) or MDD-UP (6,540 MDD-UP cases and 10,388 controls) in case–control samples of both sexes. The only significant association appeared in family-based samples (*P* = 0.01). Feng, Zhang [171] also found a high heterogeneity of the samples published in different studies.

A further meta-analysis of the same data analysed by Feng, Zhang [171] supplemented with a MDD-UP German sample [168] (7 studies) reported again no significant association of P2X7R – rs2230912 with BD either at allelic level (G allele) or at genotype level (GG + GA) (3,813 BD cases versus 6,691 controls), but a significant association with MDD-UP was found (4,182 MDD-UP cases versus 5,926 controls).

## Genome-wide association studies (GWAS)

In accordance with many candidate gene studies that did not find a significant association between the purinergic genes and major mood disorders in case–control samples of small/moderate size, the genome-wide association studies (GWAS) conducted by the Psychiatric Genomics Consortium (PGC) on very large samples (tens of thousands of subjects of different ancestries) also could not replicate the positive associations observed in some previous candidate gene studies. This was the case both for BD [Stahl, Breen [172] (20,352 cases and 31,358 controls of European descent); Mullins, Forstner [173] (BD I = 25,060 cases, 449,978 controls and BD II = 6,781 cases, 364,075 controls); O'Connell, Koromina [21] and MDD-UP (135,458 cases and 344,901 controls) [15].

But as stated by Subramanian, Tamayo [174], “Single-gene analysis may miss important effects on pathways. Cellular processes often affect sets of genes acting in concert.” This statement was confirmed by the recent GWAS of BD [20] and by a study of the Consortium on Lithium Genomics (ConLiGen) [175].

The most recent GWAS of BD [21] identified 292 loci significantly associated with the disorder passing the genome-wide significance threshold (at least *P* = 0.05^–8^) after correction for multiple testing. Many of the associated loci are expressed in the hippocampus and prefrontal cortex. No purinergic SNP or gene was among the 292 significantly associated loci in a sample of 158,036 BD cases and 2.8 million controls.

But the gene set enrichment analysis performed on the summary statistics of the polygenic risk score (PRS) computed with 6.7 million SNPs spread over the whole genome (pathway-based polygenic risk score analysis) derived from the multi-ancestry BD sample identified several genetic pathways containing purinergic receptor genes that showed a trend of association with BD (*P*-values close to GWAS significance (*P* = 0.05^_8^).

Gene set enrichment analysis is a mathematical method to predict the potential of obtaining gene set expression based on the significance of the frequency difference between two phenotypic groups (correlation with the phenotype) and considers genetic pathways [174]. The five pathways containing purinergic genes close to significant association with BD in the PGC GWAS (2025) were: GOCC-*SINAPSE* (*P2X1R; P2X3R; P2X4R; P2X5R; P2X7R; P2Y1R; P2Y4R*); GOCC-*SYNAPTIC-MEMBRANE* (*P2X1R; P2X6R; P2Y1R*); GO-BP-*REGULATION-OF-TRANS-SYNAPTIC-SIGNALING (P2X1R; P2X3R; P2Y1R; P2Y4R);* GOCC-*POST-SYNAPSE (P2X1R; P2X3R; P2X4R; P2X5R; P2X7R; P2Y1R*); GOCC-*POST-SYNAPTIC-MEMBRANE* (*P2X1R; P2Y1R; P2Y4R).* The pathways involving purinergic receptor genes are likely linked to synaptic activity, neuroinflammation***,*** and glial activation***.*** They were expressed in the prefrontal cortex and hippocampus (hippocampal pyramidal neurons, interneurons of the prefrontal cortex and hippocampus, striatum).

In spite of no significant association between BD or SCZ and specific purinergic genes in the published GWAS of the two disorders, when applying the PRS derived from the most recent GWAS of schizophrenia [176] to the prediction of psychosis in a sample of 1852 BD-I patients from Romania and UK, we found the pathway *REGULATION_OF_IMMUNE_SYSTEM_PROCESS* to be associated with the presence of psychosis (thought and perception disorders) in BD-I [177]. This pathway includes the *P2X4R, P2X7R, and P2Y12R* genes (www.GSEA UC San Diego Broad Institute). The *MITOCHONDRION* pathway also appeared associated with the presence of psychosis in BD-I patients in this Romanian-UK sample, and the same pathway was linked to the lithium treatment response in the ConLiGen study [175]; the *MITOCHONDRION* pathway contained the *P2X7R* gene. Two other pathways were linked to the treatment response to lithium in the ConLiGen sample (2367 BD patients of European ancestry, including 164 Romanian BD-I patients). These possibly associated pathways/genes were *ACETYLCHOLINE_ TRANSMEMBRANE SIGNALING RECEPTOR ACTIVITY (P2X4R*, *P2X5R, P2X6R*, *P2X7R*, *P2Y2R*, *P2Y4R, P2Y6R*, *P2Y10R*, *P2Y12R, P2Y13R*, *P2Y14R*); *CALCIUM CHANNEL PATHWAY* (*P2X1R*, *P2X4R*, *P2Y12R*).

The major affective disorders are polygenic disorders, the liability of which is influenced by many genetic loci, each with a small effect. The variability of the findings regarding the involvement of purinergic genes in major affective disorders (BD; MDD-UP) confirms the observation of a recent study that investigated the results of the available GWAS and the pathophysiological mechanisms of pharmacological treatments for psychiatric disorders [178]. The authors suggest that the genetic liability differs from the pharmacological mechanism: “Between the genes underlying the disease risk and the genes controlling the treatment response, there is only a partial overlap. Genetic architecture driving the risk for psychiatric disorders may be distinct from the pathophysiological mechanisms currently used for targeting symptom manifestations through pharmacological treatments “.

A very recent study of PGC [179] analyzed the overlap of the genetic basis of 14 psychiatric disorders, including MDD-UP and BD. The data were extracted from recent GWAS of the analyzed disorders (1,056,201 cases; 4,616,262 controls of different ancestries). Based on genetic correlations between 6 disorders and eQTL (expression of quantitative trait loci) a pleiotropic region on chromosome 12 containing 36 genes including P2X7R and P2X4R was associated with all 6 disorders (anxiety disorder, MDD-UP, BD, ADHD, schizophrenia, post-traumatic stress disorder (PTSD)) with significant genetic correlations between disorder pairs (rg from 0.55 to 0.87; *P*-values from 4.50E-07 to 5.10E-12). Moreover, the P2X7R gene was included in the hierarchical genetic factor that underlies several psychiatric disorders and may explain common variance across disorders. This finding shows that the action of purinergic genes is not disorder specific. The MDD-UP, PTSD, and anxiety disorders (internalizing factor) and the SB factor (schizophrenia and BD) showed high levels of polygenic overlap and local genetic correlation and very few disorder-specific loci.

Another factor with a major impact on the variability of the results provided by genetic association studies with respect to purinergic genes resides in the clinical heterogeneity of the samples caused by ascertainment methods (clinical, community, self-reported cases), diagnostic criteria, differences in the genetic structure of the populations, and sample size. Moreover, many SNPs in GWAS are imputed based on linkage disequilibrium, not really genotyped in the subjects. In the case of BD, the last GWAS (2025) showed that differences in ascertainment method and the BD subtype may lead to differences in the genetic architecture [20].

Here, it is important to say that GWAS studies show many limitations [180]. One of these limitations, maybe the most important, is that they are very sensitive to allele frequencies. Results may be biased if the control group has allele frequencies that are not representative of the population from which the patient population is drawn. This may explain in part the disparate findings across studies [181]. Here it is to mention also since neurologic disorders are strongly dependent on the context and the ambient conditions too, it is unlikely that a single substitution could be strongly significantly associated to a specific condition.

## Conclusions

P2X receptors play a significant role in neuroinflammation and neuroplasticity changes linked to major affective disorders. Among the different receptor subtypes, the P2X7R has been the most extensively studied. Its activation by elevated extracellular ATP during stress or tissue damage promotes neuroinflammation through the activation of the NLRP3 inflammasome and the release of pro-inflammatory cytokines such as IL-1β. Experimental studies consistently show that pharmacological blockade or genetic deletion of P2X7R produces antidepressant-like effects, highlighting its potential as a therapeutic target. Other P2X receptors may also contribute to mood regulation through distinct mechanisms, such as P2X4R is involved in microglial activation and neuroinflammatory signaling but can also influence the release of brain-derived neurotrophic factor (BDNF), suggesting a complex and context-dependent role in depression. In contrast, receptors such as P2X3 and P2X1 appear to contribute more indirectly, mainly through their involvement in chronic pain, inflammation, and peripheral physiological processes. Overall, accumulating evidence suggests that purinergic signaling, particularly through P2X receptors, represents a promising area for understanding the neurobiological mechanisms of depression and for developing novel therapeutic strategies.

## Data Availability

No datasets were generated or analysed during the current study.
